# The differentiated impacts and constraints of allometry, phylogeny, and environment on the ruminants’ ankle bone

**DOI:** 10.1038/s42003-025-07898-z

**Published:** 2025-03-18

**Authors:** Orgebin Pierre, Ilya Dziomber, Aiglstorfer Manuela, Mennecart Bastien

**Affiliations:** 1 Natural Science Collections, Martin Luther University Halle-Wittenberg, Halle (Saale), Germany; 2 Museum für Naturkunde, Leibniz- institut für Evolutions- und Biodiversitätsforschung, Berlin, Germany; 3 Institute of Plant Sciences, Université de Berne, Bern, Switzerland; 4 Oeschger Centre for Climate Research, University of Bern, Bern, Switzerland; 5 Naturhistorisches Museum Mainz/Landessammlung für Naturkunde, Mainz, Rheinland-Pfalz Germany; 6 Naturhistorisches Museum Basel, Basel, Augustinergasse 2 Switzerland

**Keywords:** Evolutionary ecology, Palaeontology

## Abstract

The astragalus is a hinged bony organ common to many tetrapods. Several factors, including allometry, phylogeny, and environment, constrain its morphology. Due to the underlying risk of these factors being confounding, previous works have frequently highlighted the difficulty in discerning the specific influence of each factor. Here, we conducted allometric and size-adjusted clade and ecomorphological analyses to assess the contribution of each of these three parameters to the morphological variation of the astragalus in ruminant artiodactyls. 3D geometric morphometric analyses confirm the astragalus’ highly integrated structure and multifactorial morphological responses. Sturdier astragali are correlated with heavier bodies. Bovids tend to display larger proximal trochlear ridges, and moschids show a prominent posterior process. The degree of development of areas where joints and ligaments intersect reflects the degree of freedom of the ankle and the locomotion type. This study provides new perspectives on the evolution of ruminants and their interactions with their environment.

## Abstract (web summary)

3D geometric morphometrics reveal how phylogeny, allometry, and locomotor adaptations shape the morphology of the ruminant astragalus, highlighting its integrated nature and evolutionary constraints.

## Introduction

Bones support loads, resist muscular contractions, and facilitate body movements^1^. Their shape is constrained by both mass and movement^1–3^. The ecological niche and phylogeny also influence bone structure^4–7^. In this context, ecomorphology aims to identify morphological characters that covary with the environment, independently of phylogeny^4^, aka it examines functional morphology (relationship between form and function) and the respective habitats^4,5,8^. In studies on extinct species, extant species with known habitat preferences are used to identify morphological convergences correlated with habitat^5^. Postcranial elements of mammals are often used with this approach, such as in carnivores^7,9^, in primates^10–12^, and in rodents^13^. Thanks to their specific and ecological diversity and their profusion in the fossil record, one of the most abundantly used models for paleoenvironmental reconstitutions are ruminant artiodactyls (e.g.^14^ on Ruminantia^4,15–20^; on Bovidae^5,21,22^; on Cervidae).

The astragalus, an element of the hock joint, is a key structure to understanding locomotion, being the hinge element between the autopod and the zeugopod^8^. Ruminants exhibit a distinctive anatomical configuration of their astragalus among mammals. While possessing the typical proximal tibial trochlea, they also feature a distal trochlea that engages with their autapomorphic fused cuboid-navicular bone^8,23^. Consequently, the astragalus in ruminants functions as a dual hinge joint between the metatarsus and the tibia. Its morphology significantly limits inversion and eversion movements, predominantly confining ankle motion to the anteroposterior direction^8,24^. This peculiar anatomical adaptation has been interpreted as a stabilization mechanism for the joint, presumably evolving an enhanced cursoriality in the earliest artiodactyls^24^.

The astragalus is an integrated bone subjected to several forces that may be concomitant^5,6,8^ such as size allometry, i.e., its size evolves proportionally to the size of the animal, which makes it an efficient proxy for body mass estimations on fossil specimens^25–27^. Moreover, while the distinction between Tragulina and Pecora based on the position of the trochlea appears relatively straightforward, the differentiation within the Pecora families presents more difficulties and lacks distinguishable apomorphic traits in the astragalus. Previous studies have pointed out the intricacies involved in this classification and the presence of multiple episodes of evolutionary convergence^28,29^. This suggests that finer distinctions within Pecora require more nuanced analyses and consideration. Due to its high compactness and its specific morphology, the astragalus of ruminants is also characterized by a high preservation potential in the fossil record, making this structure a good candidate for paleoecological studies^8^. Also, this bone is widely used to reconstruct paleoenvironments in the context of human evolution (e.g.^8,21^). However, the method’s applicability in non-African environments and the limitations of confounding factors (allometry and phylogeny) should be considered^6,8,30–32^.

Geometric morphometrics, a method for morphological quantification, has only been employed in limited phylogenetic contexts, primarily focusing on ecomorphology within ruminants. This includes postcranial elements of cervids and bovids^5,22,32^, and astragalus itself was mostly used in archaeozoological studies^33–35^. Here, we performed the first large-scale 3D geometric morphometrics analysis of the astragalus, which includes 109 taxa throughout the suborder Ruminantia and along 30 million years of their evolution to disentangle allometric, phylogenetic, and environmental signals. Each of the three factors was morphologically characterized thanks to statistical tests and discussed within a morpho-functional framework in regards to the ruminant artiodactyl evolution.

## Results

### Morphological variation assessment

**Fig. 1:**
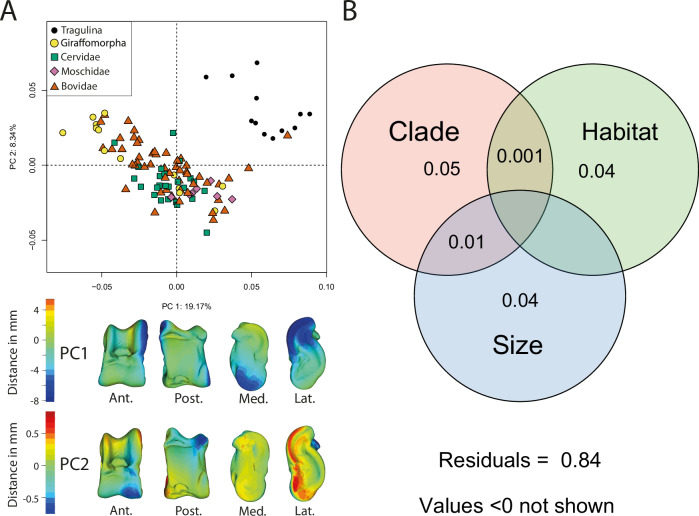
Ruminant astragalus shape variation assessment. **A** Principal component analysis (PCA) based on Procrustes coordinates including all extant and extinct ruminants. The virtual deformations along PC1 and PC2 are represented by the warped model corresponding to the mean individual deformed to reach the shape of the maximal individual on the axis. The heat maps show the regions with the most warping along positive (red) and negative (blue) scores of each PC, and light green indicates no deviation. Abbreviations: Ant. (Anterior); Post. (Posterior); Lat. (Lateral); Med. (Medial). **B** Venn diagram illustrating the variation partitioning analysis (VARPART) used on astragalus shape among extant ruminants. Values represent the adjusted R² for the size (log-transformed centroid size), the clade (assigned), the habitat (assigned categories), and the interaction of the corresponding explanatory variables.

On the principal component analysis (PCA) performed on the Procrustes coordinates of all the studied specimens, PC1 represents 19.17% of the total shape variation. Most of the variation pertains to the robustness of the bone and the degree of trochlear obliquity, in that more robust astragali exhibit less oblique trochleae (negative PC1 scores), and astragali with lower robustness display more oblique trochleae (positive PC1 scores) (Fig. 1A). PC2 represents 8.34% of the variation. Astragali on positive PC2 scores display a more developed anterolateral side and a less developed lateral ridge of the distal trochlea (LRDT) and posterior part of the medial ridge of the proximal trochlea (MRPT). On the contrary, astragali with negative PC2 scores exhibit reduced development in the anterolateral side while showing increased development in the LRDT and posterior segment of the MRPT. The scatterplot of PC1 and PC2 shows all Tragulina distributed on the positive scores of PC1 and PC2 (Fig. 1A). Pecora are grouped in the remaining morphospace, except for the insular Bovidae *Tyrrhenotragus gracillimus*, which groups with the Tragulina.

### Contribution of allometry, phylogeny, and habitat

A summary of all the following statistical results may be found in Supplementary Table 1.

Based on the ANOVA conducted on the sample comprising extant ruminants only, we observe a strong correlation between the mass (in kg) and the astragalus size, and it is predictive (*p-value* < 0.001, R² = 0.89). Based on all the ruminants (extant + extinct species), the astragalus’ size (log-transformed centroid size) exhibits a statistical correlation with its shape, whether evaluated through MANCOVA (*p-value* = 0.001; R² = 0.1) or PGLS (*p-value* = 0.001; R² = 0.03). Based on the extant ruminants only, the MANCOVA also shows a statistical correlation between astragalus size and shape (*p-value* = 0.001; R² = 0.08). However, the PGLS does not (*p-value* = 0.052; R² = 0.03). The impact of the size cannot be dissociated from the impact of the phylogeny due to sampling bias. Extant Tragulina and Moschidae are all only represented by small species, while Giraffomorpha are all large. Extinct species bring more size diversity. Nevertheless, the permutation tests of the log-transformed centroid size in the phylogenetic frame (Supplementary Fig. 1) show that the size distribution is not random along the phylogeny (*p-value* < 0.0001). Moreover, the variation partitioning analysis (VARPART) conducted on extant ruminants demonstrates that the size explains 4% of the shape variation, and clades and size jointly still explain 1% of the total morphological variation.

The permutation test applied to the PC scores within the phylogenetic framework (Supplementary Fig. 1) demonstrates a significant non-random distribution of shape across the entire ruminant phylogeny (*p-value* < 0.0001; Supplementary Table 1). The dataset encompassing Procrustes coordinates of all ruminants exhibits covariances among species that closely resemble the expected covariances under Brownian motion (Pagel’s λ = 0.74; *p-value* < 0.001). Nevertheless, we observe a high within-group variability (*K* = 0.39; *p-value* = 0.001). Following the variation partitioning analysis (VARPART) conducted on extant ruminants, clades explain 5% of the shape variation.

According to the MANCOVA, we observe a statistically significant correlation between habitat and the shape of the astragalus (*p-value* = 0.001; R² = 0.09). The PGLS does not indicate an influence of habitat on shape (*p-value* = 0.166; R² = 0.05). Then, the impact of the habitat cannot be dissociated from the impact of the phylogeny since some clades are currently restricted to specific environments, for instance, Tragulidae in tropical forests and Moschidae in the mountains. Nevertheless, following the variation partitioning analysis (VARPART) we conducted on extant ruminants, habitat and clades jointly explain only 0.001% of the shape variation, while the habitat alone explains 4% of the shape variation. Habitat and size together and the superposition of the three factors within our extant ruminant sample demonstrate no shared variation (value < 0).

### Allometric signal

**Fig. 2:**
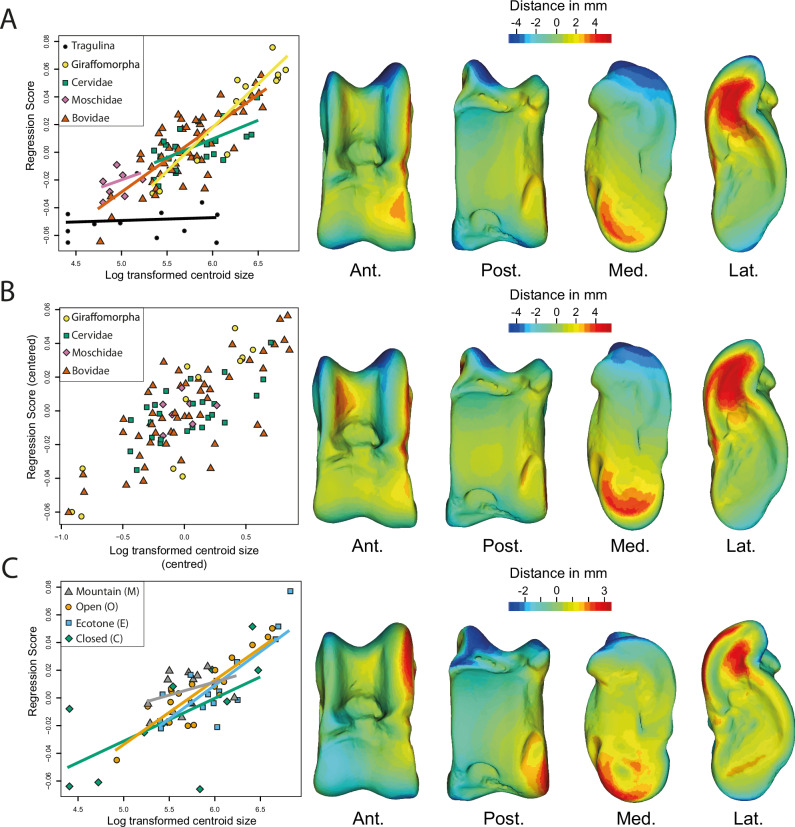
Correlation between centroid size and astragalus shape in ruminants. **A** Regression analysis based on Procrustes coordinates and including all clades, where the log-transformed centroid size is plotted against the regression score which is a projection of the data points in shape space onto an axis in the direction of the regression vector^78^. The regression lines are indicated by clade. **B** Log-transformed centroid size as a function of regression score, pooled by clade, with each clade’s centroid size distribution centered on zero with its mean. Only pecoran ruminants are included here, since Tragulina has a different allometric trend. **C** Regression analysis based on Procrustes coordinates including extant ruminants only, where the log-transformed centroid size is plotted against regression score, with regression lines indicated by habitat. The virtual deformations along the regression line are represented by the warped model corresponding to the mean individual deformed to reach the shape of the maximal individual on the axis. The colors represent the intensity of the local shape difference between extreme individuals along the regression line. Red indicates a positive deviation of the maximal individual from the minimal individual, blue indicates a negative deviation, and light green indicates no deviation. Abbreviations: Ant. (Anterior); Post. (Posterior); Lat. (Lateral); Med. (Medial).

Regression of the Procrustes coordinates (extant and extinct ruminants) on the log-transformed centroid size shows a significant correlation between astragalus size and shape (*p-value* < 0.001; Adjusted R² = 0.59). The overall appearance of astragali is more robust in large taxa and more slender in small ruminants (Fig. 2A). This is due to a lower width/length ratio of astragalus and a more medially flared distal trochlea in large taxa. As demonstrated with the permutation test (p-value < 0.0001), the regression plot shows that the size distribution within the 6 clades is not random. The smallest ruminants in the sample are Tragulina and Moschidae, while the largest are Giraffomorpha. There is a clear difference in the allometric trend between Tragulina and Pecora astragali (Fig. 2A).

Since the size and clades are not fully independent (VarPart = 0.01; Supplementary Table 1 & Supplementary Fig. 1) and the differences in slopes observed between the different Pecora clades (contrary to Tragulidae) are lower than 5% (Supplementary Table 2), pooled by clade regression of the Procrustes coordinates (Pecora only) on the log-transformed centroid size has been performed. It shows a significant correlation between astragalus size and shape (*p-value* < 0.001; Adjusted R² = 0.59). As in the previous analysis, the overall appearance of astragali is more robust in large taxa and more slender in small Pecora (Fig. 2B). Morphological changes are the same as those observed with the regression, including all ruminants. However, we do observe that in large Pecora, the medial ridge of the proximal trochlea is wider than in small ones.

The regression of the Procrustes coordinates on the log-transformed centroid size (based on extant ruminants only) shows a significant correlation between astragalus size and shape (*p-value* < 0.001; Adjusted R² = 0.56). Morphological changes are the same as those observed with the regression including all ruminants (Fig. 2C). The regression plot shows the size of astragali within each habitat category varies, and there is no consistent trend of larger or smaller astragali in any specific habitat (VarPart value < 0).

### Clade analysis

**Fig. 3:**
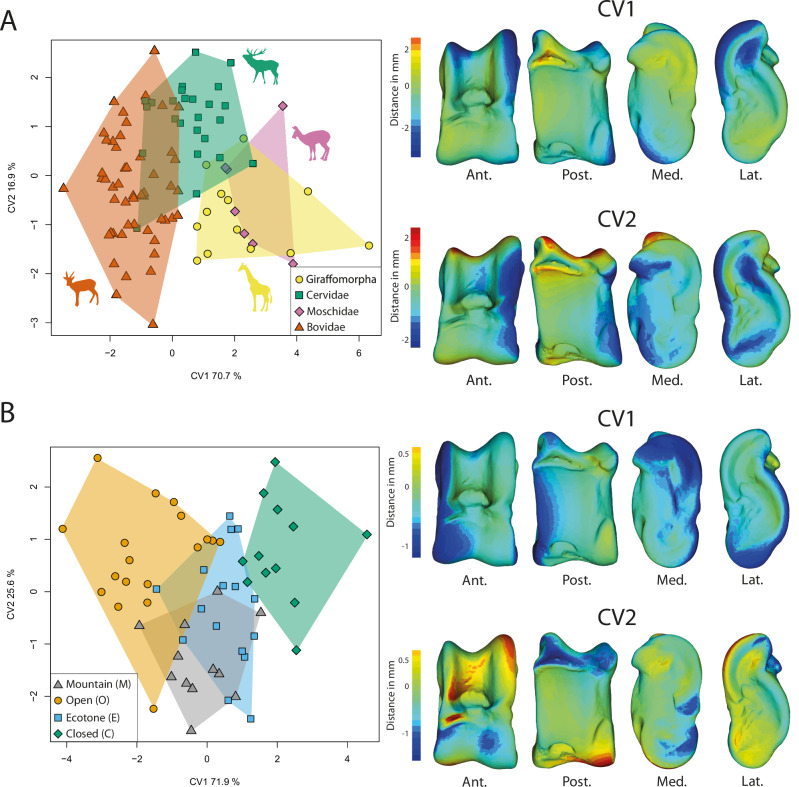
Canonical Variates Analysis (CVA) characterizing group-specific morphologies. **A** CVA on extant and extinct Pecoran ruminants to characterize differences between clades. The plot including CV3 can be found in Supplementary Fig. 3. **B** CVA on extant ruminants to characterize morphological differences between assigned habitat categories. The virtual deformations along each axis are represented by the warped model corresponding to the mean individual deformed to reach the shape of the maximal individual on the axis. The colors represent the intensity of the local shape difference between extreme individuals along the axis. Red indicates a positive deviation of the maximal individual from the minimal individual, blue indicates a negative deviation, and light green indicates no deviation. Abbreviations: Ant. (Anterior); Post. (Posterior); Lat. (Lateral); Med. (Medial). Silhouettes modified from Mennecart et al.^47^.

We used the residuals of the regression analysis based on Procrustes coordinates pooled by clades (Fig. 2B) for our clade-related discriminant analyses, since there is a covariation between the clade and the size (VarPart = 0.01; Supplementary Table 1 & Supplementary Fig. 1), to preserve clade-related shape information and remove size-related shape variation. To achieve this, we first performed a Principal Component Analysis (PCA) on the residuals of the regression, and then conducted Canonical Variates Analysis (CVA) on the first 11 principal components (49% of the total variance). The remaining variance may represent noise (Supplementary Fig. 2). Tragulina and Pecora exhibit distinct allometric trends (Fig. 2A). Therefore, we applied this size correction method exclusively to the Pecora clades. The CVA allows for the discrimination of the clades (Fig. 3A; Supplementary Fig. 3). CV1 explains 70.7% of the variation and separates Bovidae (negative scores) from other pecoran ruminants (positive scores; Fig. 3A). The astragalus of bovids has proximal trochlear ridges (MRPT and LRPT) that are more developed laterally. On CV2 (16.9%), the Cervidae are confined to the positive pole, while the other clades are distributed along the axis. The astragali of Cervidae exhibit a higher width-to-length ratio and a less pronounced posterior than negative score ruminants. CV3 (12.4%) separates Moschidae (negative scores) from Giraffomorpha (positive scores; Supplementary Fig. 3). Giraffomorpha astragali feature higher proximal trochlear ridges compared to Moschidae. In Giraffomorpha, the medial part of the plantar articular facet is shifted laterally relative to Moschidae. Additionally, the posterior process is globose in Giraffomorpha but more pronounced in Moschidae. The distal trochlea is also less developed on the medial side in Giraffomorpha. When considering reclassification rates, correct reclassification scores for the different clades are far beyond the random values, with picks of correct reclassification reached by the Bovidae (84%, see Table 1).

**Table 1:** Cross-validated classification results based on the CVA analyses performed in this study

Pecora (Clade analysis)					Extant (Ecomorphology analysis)				
Cross-validated classification results in frequencies					Cross-validated classification results in frequencies				
	Giraffomorpha	Cervidae	Bovidae	Moschidae		Closed	Ecotone	Open	Mountain
Giraffomorpha	6	3	2	2	Closed	8	4	0	0
Cervidae	1	17	7	0	Ecotone	1	9	2	4
Bovidae	2	6	43	0	Open	0	4	13	1
Moschidae	1	3	0	3	Mountain	1	2	1	8
**Cross-validated classification result in %**					**Cross-validated classification result in %**				
	Giraffomorpha	Cervidae	Bovidae	Moschidae		Closed	Ecotone	Open	Mountain
Giraffomorpha	46.15	23.08	15.38	15.38	Closed	66.67	33.33	0	0
Cervidae	4.00	68.00	28.00	0.00	Ecotone	6.25	56.25	12.5	25
Bovidae	3.92	11.76	84.31	0.00	Open	0.00	22.22	72.22	5.56
Moschidae	14.29	42.86	0.00	42.86	Mountain	8.33	16.67	8.33	66.67
**Overall classification accuracy: 71.88%**					**Overall classification accuracy: 65.52%**				
**Kappa statistic: 0.54**					**Kappa statistic: 0.54**				

### Ecomorphological analysis

As there is no covariation between habitat and size in the studied dataset (VarPart < 0.00; Figs. 1B, 2C), we directly used the regression residuals (not pooled) for the habitat-related analyses. We performed a PCA on the regression residuals. To discriminate individuals according to habitat categories, we performed a CVA on the first 8 principal components (48% of the total variance, Supplementary Fig. 2) from the PCA (Fig. 3B). The CVA allowed us to distinguish individuals based on their habitat classification. CV1 explains 71.9% of the variation and separates categories open (O; negative score) and closed (C; positive score). Other habitat categories (Ecotone and Mountain) are located between them. O category astragali have a larger medial side with pronounced medial extension and a wider lateral side of the posterior articular facet (PAF) than C category astragali. CV2 explains 25.6% of the variation. The M category on this axis is concentrated in the negative pole. O and C categories are mostly found in the positive scores, while the E category partially overlaps with the mountain category. The astragali at the negative pole have a wider medial side of the distal trochlea than those positioned at the positive pole. The negative pole astragali have a wider insertion area of the talofibular ligament (LA2) with a more pronounced posterior process than other astragali. When considering reclassification rates, correct reclassification scores for the different environments are beyond the random values, with picks of correct reclassification reached by the open category (72%, Table1).

## Discussion

### Influence of allometry

**Fig. 4:**
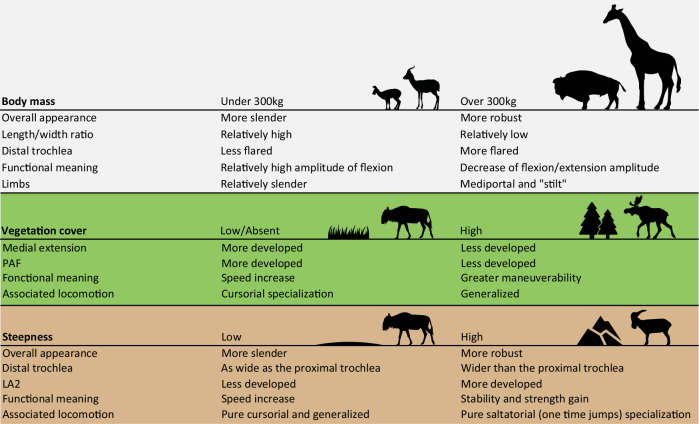
Summary of astragalus morphological trends associated with mass, vegetation cover, and the degree of steepness of the ruminant environment. References for the silhouettes used here can be found in the reference section. Gazella gazella by Rebecca Groom (CC BY 3.0) - PhyloPic; Bos bison by Lukasiniho (CC BY-NC-SA 3.0) - PhyloPic; Connochaetes taurinus by Lukasiniho (CC BY-NC-SA 3.0) - PhyloPic.

Using linear measurements, studies have demonstrated the interest of the astragalus in ecomorphology within Bovidae^4,15,19^. However, Barr^8^ not only pointed out the fact that the metrics used in these studies do not fully characterize the complex geometry of some astragalus portions, like the trochlea but also highlighted that most of the measurements examined are highly correlated with body size, in line with the findings of Klein et al.^30^ We also found that the size of the astragalus and the mass of the ruminants are correlated (Supplementary Fig. 4) and have a strong influence on the shape of this bone (Fig. 2). This is in line with the results of Martinez and Sudre^25^. However, based on our sample, the morphological changes are not progressive and a critical mass seems to exist, following the trend observed in mammal limbs by Biewener^36^. In most ruminant species above 300 kg, the width/length ratio of astragalus is lower (Figs. 2, 4). This feature gives the astragalus of these taxa a more robust overall appearance. Presumably, this helps the bones to resist a higher mass by distributing forces over a larger surface. Bones have proportionately larger epiphyses in heavy species^3,32^. This would support larger facet joints for better force dissipation. Another possible effect, surely concomitant with the previous one, is that it could help bones resist bending stress^32,36^. Indeed, Gambaryan^37^ associates the medial-support locomotion mode with a reduction in the flexion amplitude and extension of the crurotarsal joint. Thus, by decreasing the moment at which the joint flexes, the forces involved are lower^32^.

This model seems applicable to most extant ruminants in our sample, except for *Giraffa camelopardalis*. Species of this genus are characterized by high limb heights, despite masses reaching 2000kg in some male individuals^38,39^. Gambaryan^37^ attributes a locomotor type of its own to *Giraffa*: the “stilt” type. This type is characterized by an increase in the power developed during the support phase^37^. Furthermore, Basu and Hutchinson^40^ showed that *Giraffa camelopardalis* exhibits a lower effective mechanical advantage (EMA) than *Sivatherium giganteum*, due to limitations in their musculoskeletal structure. This inadequacy prevents them from sustaining the expected trend in EMA observed in animals weighing up to 300 kg.

### Influence of the phylogeny

The astragalus is often used to define characters in ruminant phylogenetic analyses. Indeed, the astragalus is a key feature to distinguish Tragulina from Pecora; the first group is characterized by a more oblique trochlea compared to the second^28,41–45^. This should be considered in parallel to a different allometric trajectory between Tragulina and Pecora, indicating different trends in morphologies (Fig. 2A). Interestingly, Van der Geer^46^ observed an oblique trochlea in *Myotragus* and *Hoplitomeryx*, two insular pecoran ruminants. Van der Geer^46^ argues that the convergence of the zeugopod, due to the obliquity of the astragalus trochlea, compensates for the divergence of the stylopod, itself due to a wider abdomen in island forms.

**Fig. 5:**
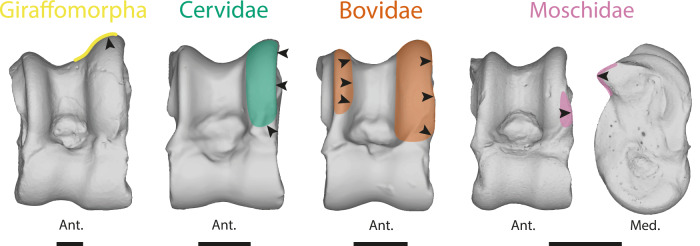
Astragalus morphological trends are observed in clades of Pecora in this study. Moschidae are characterized by their posterior process more prominent and their LRPT’s peculiar morphology with its central part wider laterally. Bovidae tend to display larger proximal trochlear ridges than other Pecora. Giraffomorpha exhibit a higher LRPT. Cervidae in our sample have in general thinner lateral ridge of proximal trochlea than other Pecora. The species used to represent each clade are: Giraffomorpha, *Palaeomeryx eminens* (NMB 209); Cervidae, *Dama dama* (NMB 1641500); Moschidae, *Moschus moschiferus* (ZFMK 664); Bovidae, *Antidorcas marsupialis* (NMB 10853). Abbreviations: Ant. (Anterior); Post. (Posterior); Med. (Medial). The scale bar represents 1 cm.

Likely due to multiple occurrences of evolutionary convergence and significant intra-clade variation, distinguishing between Pecora clades based on the astragalus poses greater challenges ^(28, this study)^. Indeed, despite finding significant morphological differences between clades, most of these characters can only be observed through discreet morphometrics (Fig. 3B). Following our results, the astragalus of Bovidae is the most distinctive among Pecora. This can be explained by their more recent cladogenesis^47–49^ and their astragalus morphology being more derived than the other Pecora. Indeed, the term “advanced” Pecora is frequently used to describe the Bovidae^28^. However, considered “derived” traits in their astragalus seem to characterize ruminants living in open environments (Figs. 3; 4^8,28^;). Therefore, size and mass should also be taken into account. Indeed, the astragali of Giraffomorpha in our sample are more robust thanks to a more flared distal trochlea. These traits are also observed in large Bovids and are associated with an increase in size and mass (Figs. 2, 4). Nonetheless, neutral evolution can take place on structures free of physical and mechanical constraints, and provide essential information for phylogeny^47,50^. Bovidae tend to display larger proximal trochlear ridges than other Pecora (Fig. 5), while Moschidae have a more prominent posterior process and their LRPT exhibits a peculiar morphology with its central part wider laterally (Figs. 3A, 5). It is interesting to note that despite the closer phylogenetic relationship between these two families, their astragali cannot be confounded (Table 1). The identification of traits in astragalus of Cervidae and Giraffomorpha becomes more difficult, as our results show that the morphological changes are more subtle and not restricted to particular areas. These clades of Pecora first derived within the crown group and diverged within a short time^51^. They may retain a more ancestral common astragalus morphology. Nevertheless, we can observe in our sample that the LRTP is higher in Giraffomorpha and laterally thinner in Cervidae.

### Influence of the habitat

Ecomorphology is considered a taxon-independent approach, as it can be employed with mammalian remains that have not been identified beyond the family level^22,52^. The current ecological diversity of the ruminant families does not adequately represent overall past diversity^53,54^. Today, Bovidae are the most diversified ruminant family^38^ and are a widely used model for paleoenvironmental reconstructions^4,15–20^. However, Rozzi and Palombo^31^ highlighted some inadequacy of the method introduced by DeGusta and Vrba^4^ when not applied to African bovids. Even if our extant ruminant sample prevented us from being fully taxon-independent we can observe shared morphological trends that may be linked to environmental constraints (Fig. 3B, Fig. 4^29^;). We observe a gradation between the different habitats, along a continuum from open to closed habitat (Fig. 3B). In habitats with low vegetation cover and few hiding places, speed is the main strategy against predation^8,14,37^. In closed habitats, where ground obstacles are frequent (56), maneuverability is more important than speed (Fig. 3^5,8,14,15,19,32,37^;). We observe a greater development of medial extension in environments with lower or no vegetation (Fig. 3B). This is consistent with the fact that in cursorial forms, a decrease in joint mobility outside the parasagittal plane contributes to avoiding the risk of dislocation during running^8,37,55^. Presumably, having a more developed medial extension in these taxa creates a conduit helping to channel the tibia, which may serve to optimize parasagittal movement.

**Fig. 6:**
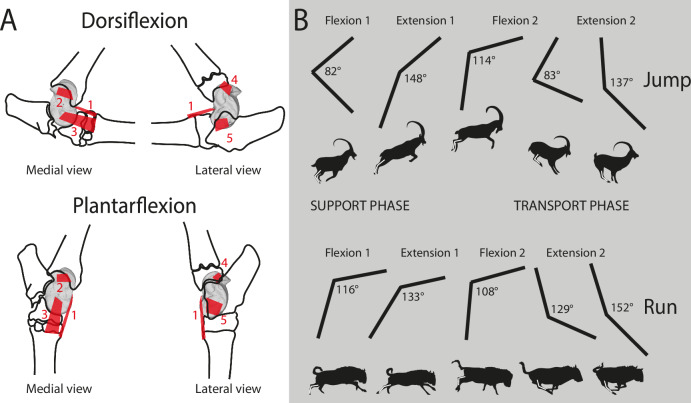
Ligaments of astragalus and phases and periods of crurotarsal joint activity. **A** Positions of astragalus bone ligaments^8,56,60^. dorsal (1); talotibial (2); talometatarsal (3); talofibular (4); talocalcaneal (5). Two stages of the crurotarsal joint are illustrated here: dorsiflexion and plantarflexion. **B** Phases and periods of crurotarsal joint activity^37^. Phase of support: preparatory period (Flexion 1); starting period (Extension 1). Phase of free transit: drawing-up period (Flexion 2); adjustment period (Extension 2). Two modes of locomotion are illustrated here: One-time jumping and running. Values of the crurotarsal joint angle are indicated for each period. Figure 6. Sindh Ibex jumping on the Mountain - YouTube; Great Migrations Wildebeest GIF by Head Like an Orange - Find & Share on GIPHY.

Another continuum may be observed when considering the topography of the habitat as a contributing parameter (Fig. 3B). Thus, species inhabiting steep and mountainous environments (M) appear to exhibit distinct morphological traits compared to other species (Figs. 3B; 4). In these taxa, there is an increased sturdiness of the astragalus, due to a widening of the distal trochlea. We also observe a tendency towards an augmentation of the surface area occupied by the LA2 (Figs. 3B, 4). We can assume that a wider LA2 implies an accentuated development of the talofibular ligament. This ligament is dorsal to the axis of the talocrural joint (Fig. 6A). It is therefore stretched by the flexion of this joint and tends to limit its movement^56^. This may help avoid the risk of dislocation during joint flexion. This function is similarly supported by the greater development of the posterior process and the deepest PTF, which further reinforces the stability. Gambaryan^37^, associates ruminants living in topographically complex environments with purely saltatorial locomotion and requires greater strength to be developed during the support phase (ref. ^37^, Fig. 6B). We suppose that the morphology of the astragalus of M species is a potential response to the steep gradient of mountainous/rocky environments. The increased strength developed when taking off would enable longer, more precise jumps to move from rock to rock^37^.

## Methods

### Material and data acquisition

Institutional abbreviations: GSPY, Geological Survey of Pakistan and Yale Peabody Museum, MNHN, Muséum National d’Histoire Naturelle, Paris, France; NHMUK, Natural History Museum of United Kingdom, London, United Kingdom; MNCN, Museo Nacional de Ciencas Naturales, Madrid, Spain; MHNBe Naturhistorisches Museum Bern, Bern, Switzerland; NMB, Naturhistorisches Museum Basel, Basel, Switzerland; MGL, Musée cantonal de géologie de Lausanne, Lausanne, Switzerland; NHMMZ, Naturhistorisches Museum Mainz, Mainz, Germany; SMNS, Staatliches Museum für Naturkunde Stuttgart, Stuttgart, Germany; ZFMK, Museum Koenig Bonn, Bonn, Germany.

**Fig. 7:**
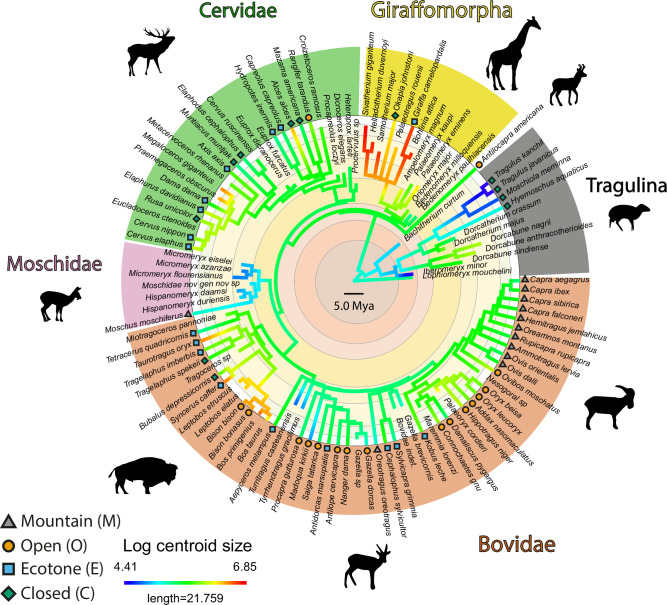
Phylogeny of ruminants studied. The log centroid size of the astragalus (a proxy for the body size) and the assigned habitat categories (for extant taxa) are also indicated. The topology used here is based on multiple previous works available in Supplementary Data. The package phytools (version 2.0^73^) was used to plot centroid size values on the phylogeny. Silhouettes modified from Mennecart et al.^47^.

The sample comprises 205 astragali belonging to 58 extant species and 51 extinct species of ruminants located in several different institutions (Fig. 7). We selected specimens intending to cover as much of the group’s diversity as possible in terms of size, phylogeny, and habitat (Fig. 7). The nodes of the main clades are calibrated in time by the fossil record according to Mennecart et al.^47^ In order to quantify intraspecific variation, some species are represented by several individuals (*Alces alces*, *Antilocapra americana, Axis axis, Bachitherium curtum, Bedenomeryx milloquensis, Bedenomeryx paulhiacensis, Bohlinia attica, Bos primigenius, Bos taurus, Capra aegagrus, Capra ibex, Capra sibirica, Capreolus capreolus, Cervus elaphus, Croizetoceros ramosus, Dicroceros elegans, Dorcatherium crassum, Elaphodus cephalophus, Eucladoceros ctenoides, Gazella dorcas, Helladotherium duvernoyi, Hispanomeryx daamsi, Hyemoschus aquaticus, Iberomeryx minor, Moschidae nov gen nov sp, Megaloceros giganteus, Metacervoceros rhenanus, Micromeryx azanzae, Micromeryx eiselei Micromeryx flourensianus, Miotragoceros pannoniae, Moschiola meminna, Moschus moschiferus, Muntiacus muntjak, Okapia johnstoni, Oriomeryx major, Ovis dalli, Ovis orientalis, Palaeoryx cordieri, Palaeomeryx eminens, Palaeomeryx kaupi, Rangifer tarandus, Saiga tatarica, Samotherium major, Sivatherium giganteum, Tetracerus quadricornis, Tyrrhenotragus gracilimus*).

We generated the 3D models using different technologies (see Supplementary Data for details). As our study centered on the external morphology of the talus, the impact of utilizing different technologies is minimal^57^. 143 individuals were surface-scanned with an Artec Space Spider surface scanner and reconstructed on Artec Studio 17. Two specimens from MNHN were digitized with an Aicon Smart Scan at Plateau de Morphométrie du Muséum MNHN, CNRS UMS 2700 OMSI. Four specimens were surface-scanned using a FARO Laser ScanArm and digitized on Faro RevEng®2022.3 software. 33 individuals (ZFMK, SMNS) were scanned with x-ray computed tomography at the Staatliches Museum für Naturkunde Stuttgart using a Bruker Skyscan 1272 equipped with a 20-100 kV / 10 W x-ray source. 23 individuals were digitized using a nanoCT system nanotom (phoenix X-ray, GE Sensing and Inspection Technologies GmbH, Wunstorf, Germany) hosted at the Department of Biomedical Engineering, University of Basel. Additionally, four ct-scanned individuals from the Yale Peabody Museum were added to the sample. Details of the origin and digitization method of each individual can be found in Supplementary Data file. 3D models from CT data were reconstructed using AVIZO 9.0 software (Visualization Sciences Group). 3D data that were too large (up to 25 GB for some models derived from CT scans) were reduced with MeshLab® 1.3.3 software^58^ to 20 MB or less. Left astragali were preferentially selected. Right astragali were reversed using Landmark Editor® 3.0.0.6^59^. Scale normalization in mm was performed using MeshLab® 1.3.3 software.

### Nomenclature

**Fig. 8:**
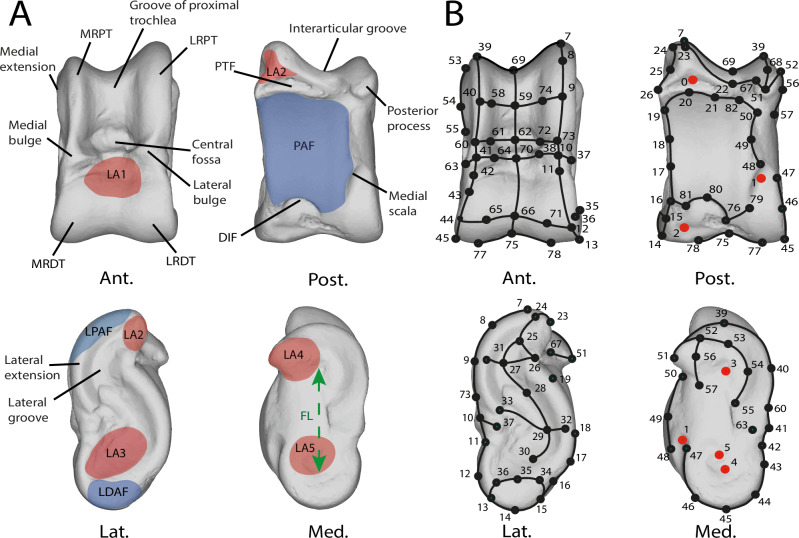
Nomenclature and landmarking procedure. **A** Nomenclature of the left astragalus of *Muntiacus muntjak* SMNS 1499. Modified from Alexander and Bennett^56^, Barone^23,60^, Haruda et al.^34^, Barr^8^, Solounias and Danowitz^61^, Pöllath et al.^35^ Blue areas represent facet joints and red areas are ligament insertion areas. **B** Landmarking procedure based on the astragalus of *Muntiacus muntjak* SMNS 1499. Numbers correspond to the order in which landmarks are placed. Red points are isolated and fixed landmarks. Black acts as anchors for the curves. Curves are made up of 10 semi-landmarks. Abbreviations: Ant. (Anterior); Post. (Posterior); Lat. (Lateral); Med. (Medial); CDT (circle of distal trochlea); CPT (circle of distal trochlea); LDAF (lateral distal facet joint); LPAF (lateral proximal facet joint); PAF (plantar articular facet); DIF (distal intracephalic fossa); PTF (proximal triangular fossa); FL (functional length); LRPT (the lateral ridge of proximal trochlea); MRDT (the medial ridge of the distal trochlea); LA1 (insertion area of the dorsal ligament); LA2 (insertion area of the talofibular ligament); LA3 (medial talocalcaneal ligament insertion area); LA4 (talotibial ligament insertion area); LA5 (lateral talometatarsal ligament insertion area). See Supplementary Data for landmarks data.

The nomenclature used for astragalus morphology (Fig. 8A) is derived from previous studies^8,23,34,35,56,60,61^.

### Ecological variables

Two ecological variables were taken into account, in the selection of extant species: mass and habitat. Mass values and habitats were taken from Kingdon^39^, Nowak^38^, and Huffman^62^. Where a range of masses was provided, the mean value for the species was used, according to other studies^4,5,32^. In our sample of extant ruminants, the mass range extends from 1.85 kg (*Tragulus kanchil*) to 1240 kg (*Giraffa camelopardalis*). The species were classified according to their habitat preferences into 4 categories (Fig. 7), based on those of Köhler^14^: closed environments (C: dense forests, high humidity), ecotones (E: transition zones with high variability of vegetation cover and humidity), open environments (O: grasslands, hot and cold deserts), mountainous environments (M: steep and/or rocky areas, with variable vegetation cover).

### Landmarking

To analyze the morphology of astragalus, we used 3D geometric morphometrics to quantify the shape of each individual. The regions of interest used to establish the protocol were defined based on characters highlighted or used in previous studies^8,34,35^. Additional characters were identified (e.g. medial extension; Supplementary Data). To capture the astragalus shape, 6 fixed landmarks were also placed (Fig. 8B) using Landmark Editor® 3.0.0.6^59^. Due to the complex, curved, and rounded shape of the astragalus, we also used a 3D sliding-semilandmarks procedure^63,64^ using R package *geomorph* (version 4.06^65,66^). Semilandmarks slide along predefined curves and surfaces while minimizing binding energy^63^. In total, the landmarking protocol is composed of 42 semilandmarks curves, of 10 semilandmarks each. The coordinates were then extracted in NTsyslandmark points (.dta) format.

### Statistics and reproducibility

**Fig. 9:**
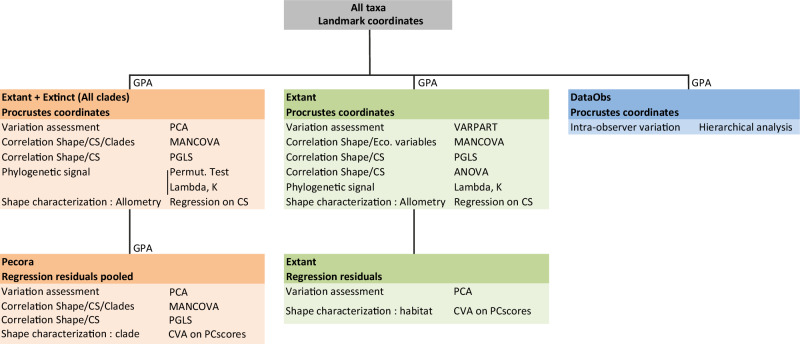
Datasets and statistical analysis performed in this study. DataObs is composed of 20 individuals (procrustean coordinates) from *Micromeryx flourensianus*: 10 replicas of the individual NMB 1122 (Steinheim am Albuch) and 10 different individuals in order to quantify intra-observer variation. Canonical variates analyses are based on 11 (Pecora) and 8 (Extant) principal components. Abbreviations: GPA (Generalized Procrustes analysis); PCA (Principal component analysis); VARPART (Partitioning analysis of variation); CS (Centroid size); MANCOVA (multivariate analysis of covariance); PGLS (Phylogenetic generalized least squares); ANOVA (Analysis of variance); CVA (Canonical variates analysis); BgPCA (Between group principal component Analysis).

All the following analyses were carried out using MorphoJ® 1.06b^67^ and R (version 4.2.2^68,69^), based on the code used by Mennecart et al.^47^ Statistical tests were used with a significance level of 5%. To compare the different morphologies of the astragalus, a generalized Procrustes analysis (GPA) was performed using the “*gpagen*” function in the *geomorph* R package (version 4.0.7; Fig. 9). This is based on three operations: configuration of coordinate scaling, translation, and rotation relative to a reference shape determined by the analysis^70^. A centroid size for each individual is obtained at the end of this analysis.

To quantify intra-observer variability on landmark positioning (DataObs), individual NMB 1122 of the species *Micromeryx flourensianus* from the Steinheim am Albuch locality, was replicated 10 times over a week (Fig. 9). The Procrustes coordinates of the replicas were then compared with those obtained on other individuals belonging to the species *Micromeryx flourensianus* ct- and surface-scanned using a hierarchical analysis performed with the “Cluster analysis” tool and the “Euclidean similarity” option on the PAST® 2.17c software^71^. The results show that intra-observer variation is lower than intra-specific variation (Supplementary Fig. 5).

Considering the large phylogenetic scale of this study, we used mean values per taxa obtained from the GPA (Procrustes coordinates and centroid size) for all following analyses. This approach was adopted to mitigate the impact of intra-specific variation over inter-specific variation. We conducted analyses on two distinct sub-samples: one comprising all taxa and the other including only extant ruminants. This differentiation aligns with the aims of our study, which involve clades and ecomorphological analyses.

On all taxa, we performed a principal component analysis (PCA; *geomorph* function “*gm.prcomp*”) to represent overall variation and evaluate the impact of allometry (Figs. 1A, 9). To test the presence of a phylogenetic signal in this dataset, we conducted a permutation test on PC scores (for shape) and log-transformed centroid size (for size) using the phylogenetic tree using MorphoJ^67,72^. Additionally, we calculated Pagel’s λ and Bloomberg’s K phylogenetic signal values using the “*phylosig*” function in the R package *phytools* (version 2.0^73^). We tested the correlation between shape and both size (log-transformed centroid size) and clades with a multivariate analysis of covariance (MANCOVA; *geomorph* function “*procD.lm*”^74^;). We also conducted a Phylogenetic Generalized Least Squares (PGLS) to investigate the influence of size on shape within a phylogenetic framework (*geomorph* function “*procD.pgls*”^75^).

On extant taxa, we carried out a partitioning analysis of variation in astragalus morphology (Fig. 1B^76^). The factors included in the model are size (Log-transformed centroid size), clades (for phylogeny), and habitat categories (for environment). To partition shape variation according to these classifiers, we used the “*varpart*” function from the package *vegan* (version 2.4-6^77^). To test the correlation between shape and both size (log-transformed centroid size) and habitat (assigned categories), we conducted a MANCOVA and a PGLS on Procrustes coordinates of our extant taxa dataset, considering both mass (log-transformed) and habitat (assigned categories). To test the predictive power of astragalus size on ruminant body mass, we used an analysis of variance (ANOVA) on log-transformed mass and log-transformed centroid size.

To visualize the morphological influence of size in all the datasets, we employed a multivariate regression model^78^ in MorphoJ. This model involved regressing the variables describing the shape of each astragalus (represented as Procrustes coordinates) against the log-transformed centroid size.

As initial analyses revealed an impact of size on the shape of datasets, a size correction was required to characterize morphologies across habitats and clades. For habitats, we directly used the residuals from the multivariate regression. However, the size distribution is not random between clades. Consequently, a pooled within-group regression utilizing log-transformed centroid size was performed on the Procrustes coordinates in MorphoJ. As defined by Klingenberg^78^, pooled within-group regression utilizes shape and size deviations of each specimen from the averages of its respective clade to compute variances and covariances. This approach ensures that the analysis focuses on within-clade variation rather than overall means. Working with residual values enables exploration of the dataset with non-allometric variation, allowing a focus on phylogenetic parameters^78^. Yet a fundamental assumption in pooled within-group regression is that all groups exhibit common allometry, meaning that the regression coefficients remain consistent across all groups (ref. ^78^; Supplementary Table 2). Tragulina and Pecora demonstrate clear distinct allometric trends. So, we opted to implement this size correction method exclusively within the Pecora clades where differences are lower than 5% (Supplementary Table 2).

To visualize the shape differences among clades of Pecora and habitats in a reduced-dimensional space, we conducted between-group principal component analyses (bg-PCA, see Supplementary Fig. 6) and canonical variate analyses (CVA). Both bg-PCA and CVA provide additional insights^47,79^. The bg-PCA specifically calculates variance between groups (clades or habitats) without standardizing the variance within each group. CVA maximizes the separation of means between groups relative to the variation in the ratio of groups according to a specified grouping variable. To facilitate dimensionality reduction, CVA were applied to eigenvectors derived from a PCA performed on size-adjusted residuals, with habitats unpooled and pooled by clades (Fig. 9; ^80^). The eigenvectors selected are beyond the noise level and represent 48% (habitats) and 49% (clades) of the total variance (Supplementary Fig. 2;^81^).

We calculated the bg-PCAs using the “*groupPCA*” function in the *Morpho* package (version 2.11^82^). We performed CVAs with the “*cva*” function, from the *Morpho* package. To test the performance of the classification model, the analysis was cross-validated using the Jackknife method.

Thin Plate Splines (TPS) as described by Klingenberg^83^ were utilized to visualize our analysis results. Specifically, the mean shape derived from GPA was projected onto the specimen closest to this mean. Subsequently, this mean-shaped model underwent deformation using TPS to match the extreme shapes resulting from our different analyses (PCA, multivariate regression, CVA). Color maps were then applied to the theoretical bones to illustrate local shape deviations with the “*meshDist”* function from the *Morpho* R package.

### Reporting summary

Further information on research design is available in the Nature Portfolio Reporting Summary linked to this article.

## Supplementary information

Supplementary Information

Supplementary Data

Description of Additional Supplementary Materials

Reporting Summary

## Data Availability

Supplementary Figs. as well as the references used for the topology of the phylogeny and the results of statistical tests are available in the Supplementary Information pdf file. The detailed lists of individuals and the landmarking protocol can be found in the Supplementary Data file. All materials from which shape data are generated are housed in museum collections. The source data is available in the Zenodo repository 10.5281/zenodo.14961512^69^. 3D reconstructed models of astragali are either published and open access in MorphoMuseuM (https://morphomuseum.com/) or will be. The not yet published models are available from the corresponding authors upon reasonable request.

## Code availability

The codes generated to conduct this study is available in the Zenodo repository 10.5281/zenodo.14961512^69^.
