# Supplementary material for: The differentiated impacts and constraints of allometry, phylogeny, and environment on the ruminants’ ankle bone
Source: Commun Biol. 2025 Mar 18;8:456. doi: 10.1038/s42003-025-07898-z (PMC11920208; doi:10.1038/s42003-025-07898-z)
Supplement: Supplementary file 1 — Supplementary Information [file 42003_2025_7898_MOESM1_ESM.pdf]

Supplementary Information

The differentiated impacts and constraints of allometry, phylogeny, and environment on the ruminants' ankle bone

Orgebin Pierre[1,2], Dziomber Laura[3,4], Aiglstorfer Manuela[5] & Mennecart Bastien[6]

[1] Natural Science Collections, Martin Luther University Halle-Wittenberg, Halle (Saale), Germany.

[2] Museum für Naturkunde, Leibniz- institut für Evolutions- und Biodiversitätsforschung, Berlin, Germany.

[3]Institute of Plant Sciences, Université de Berne, 3013 Bern, Switzerland

[4]Oeschger Centre for Climate Research, University of Bern, 3012 Bern, Switzerland

[5]Naturhistorisches Museum Mainz/Landessammlung für Naturkunde Rheinland-Pfalz,55116 Mainz, Germany

[6]Naturhistorisches Museum Basel, Augustinergasse 2, 4001 Basel, Switzerland

Supplementary Table 1. MANCOVA and PGLS performed in this study.

**Extant**			p	R^2	F
**MANCOVA**	Procrustes coords	Habitat	**0.001**	0.09	1.93
		Log Centroid Size	**0.001**	0.08	5.06
		Interaction	**0.028**	0.06	1.3
**PGLS**	Procrustes coords	Habitat	0.166	0.05	0.95
		Log Centroid Size	0.052	0.03	1.7
		Interaction	0.135	0.06	1.14
**Extant + Extinct**			p	R^2	F
**MANCOVA**	Procrustes coords	Clade	**0.001**	0.13	14.4
		Log Centroid Size	**0.001**	0.1	4.5
		Interaction	**0.001**	0.06	2.2
**PGLS**	Procrustes coords	Log Centroid Size	**0.001**	0.03	2.8
**Pecora**			p	R^2	F
**MANCOVA**	Procrustes coords	Clade	**0.001**	0.06	13.1
		Log Centroid Size	**0.001**	0.1	2.3
		Interaction	**0.012**	0.03	1.4
**PGLS**	Procrustes coords	Log Centroid Size	0.181	0.03	2.7

Comment on the PGLS and MANCOVA results for Pecora: The PGLS takes global phylogeny into account by treating evolution as a continuous process and correcting for phylogenetic dependencies. In contrast, MANCOVA directly examines the effect of size while considering clades as distinct factors. Including clades in MANCOVA allows capturing variations specific to each phylogenetic group, thus highlighting the effect of size in certain groups that the PGLS model does not capture as easily. This suggests that, without considering clades, the size-shape relationship may be masked by the global phylogenetic structure (which PGLS attempts to model), but when clades are specified, the effect of size becomes clearer in particular groups. In our sample, some clades exhibit greater diversity (Bovidae) in terms of size than others (Moschidae).

Supplementary Table 2. Pairwise tests to compare regression slopes among clades of Pecora. The results indicate that Giraffomorpha and Bovidae – which exhibit comparable ranges of centroid size – do not have significantly different allometric slopes (*p.values* > 0.05). Furthermore, the significant differences observed between other clades do not exceed 5%, supporting the application of pooled per-clade regression methods, as detailed in the Methods section of the main text.

Clade 1	Clade 2	Slope Difference	*p*
Cervidae	Bovidae	0.0186	0.027
Giraffomorpha	Bovidae	0.0149	0.118
Moschidae	Bovidae	0.0352	0.031
Bovidae	Cervidae	0.0186	0.04
Giraffomorpha	Cervidae	0.0334	0.00801
Moschidae	Cervidae	0.0166	0.169
Bovidae	Giraffomorpha	0.0149	0.0991
Cervidae	Giraffomorpha	0.0334	0.01
Moschidae	Giraffomorpha	0.05	0
Bovidae	Moschidae	0.0352	0.037
Cervidae	Moschidae	0.0166	0.149
Giraffomorpha	Moschidae	0.05	0.001

A

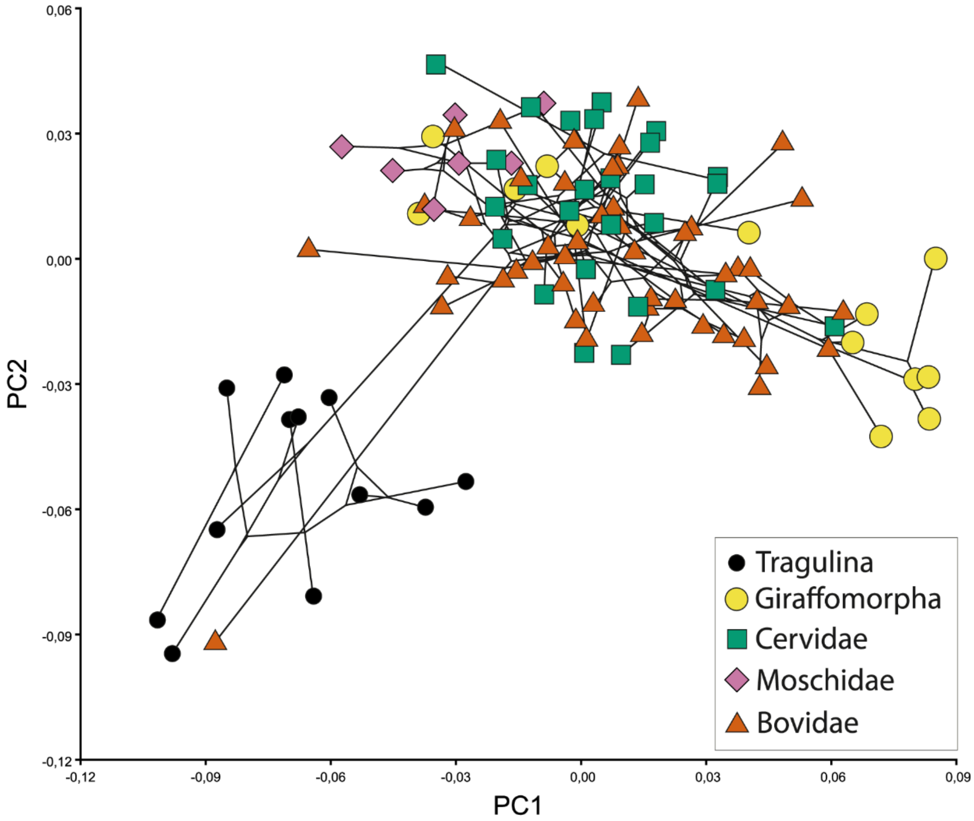

Permutation test against the null hypothesis of no phylogenetic signal
Number of randomization rounds: 10000
P-value: <.0001

B

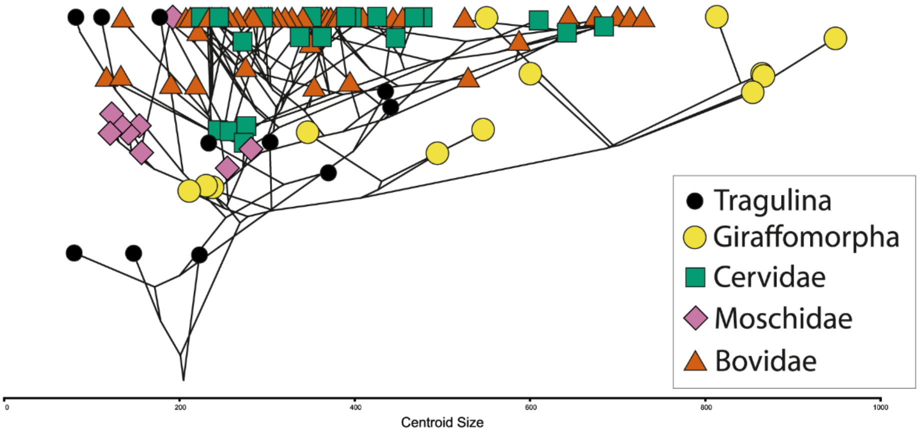

Permutation test against the null hypothesis of no phylogenetic signal
Number of randomization rounds: 10000
P-value: <.0001

Supplementary Figure 1. Permutation test on phylogeny. (A) Based on PC scores. PC1 and PC2 represent 19.17% and 8.34% respectively of the total variation. (B) Based on centroid size. Mean coordinates and centroid size values per species are used.

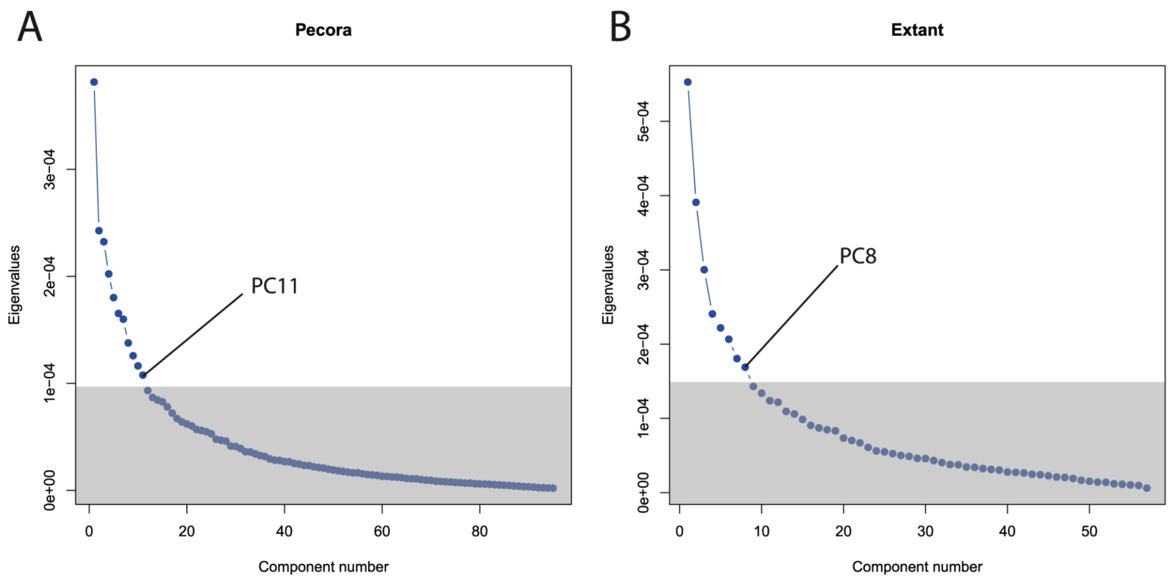

Supplementary Figure 2. Eigenvalues (represent the amount of variance in each component) of the PCAs performed on allometry-corrected residuals (mean Procrustes coordinates per species used) based on (A) extant and extinct Pecora and (B) extant ruminants only. The number of components selected for the CVA to characterize differences between clades is 11 and 8 for habitat categories. The PC representing noise is indicated with the grey area.

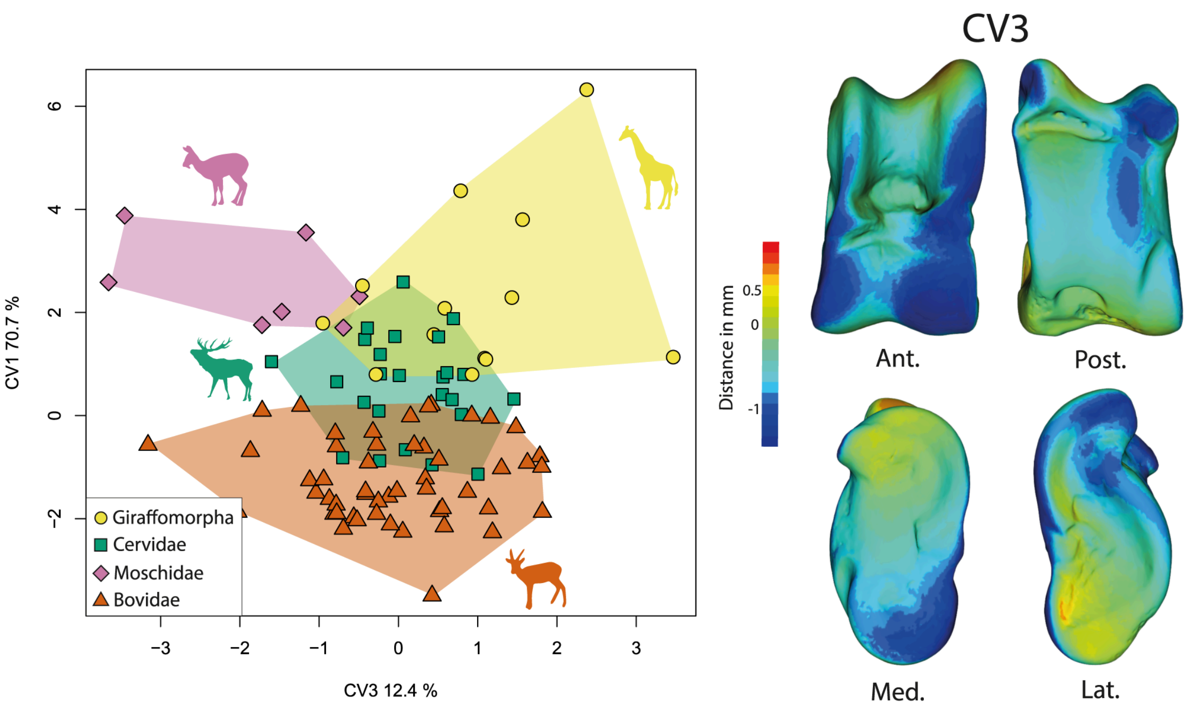

Supplementary Figure 3. CVA on extant and extinct pecoran ruminants to characterize differences between clades. Mean Procrustes coordinates per species are used.

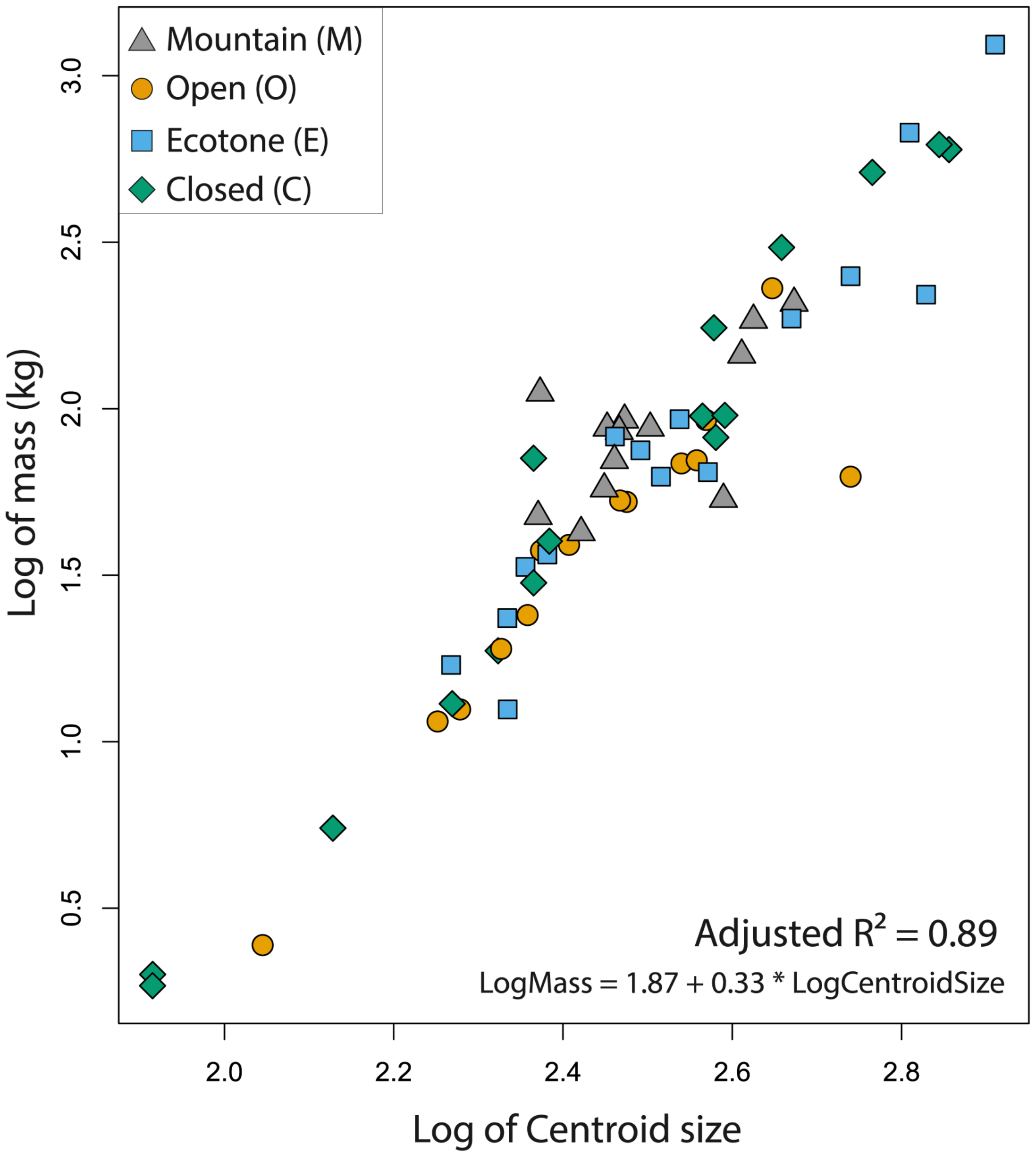

Supplementary Figure 4. Regression between the logarithm of centroid size and the logarithm of body mass. The equation is also indicated. Mean values per species are used.

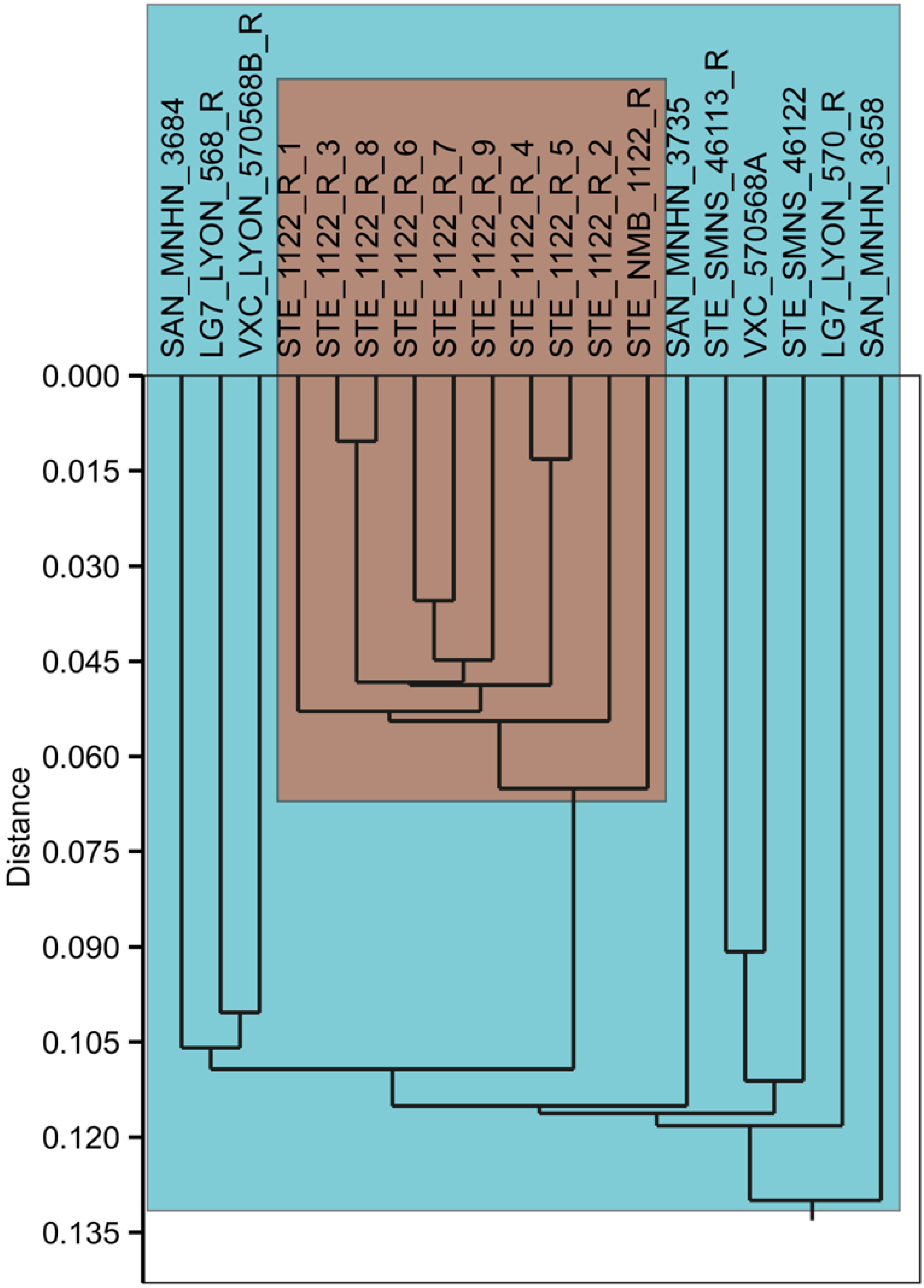

Supplementary Figure 5. Result of hierarchical analysis on the DataObs dataset. Intra-observer variation is shown in red. Intra-specific variation is shown in blue. Intra-observer variation is lower than intra-specific variation. Locality abbreviations: SAN Sansan; LG7 La Grive 7; STE Steiheim am Albuch; VXC Vieux Collonges. The letter R in the name of some individuals indicates that they have

been mirrored.

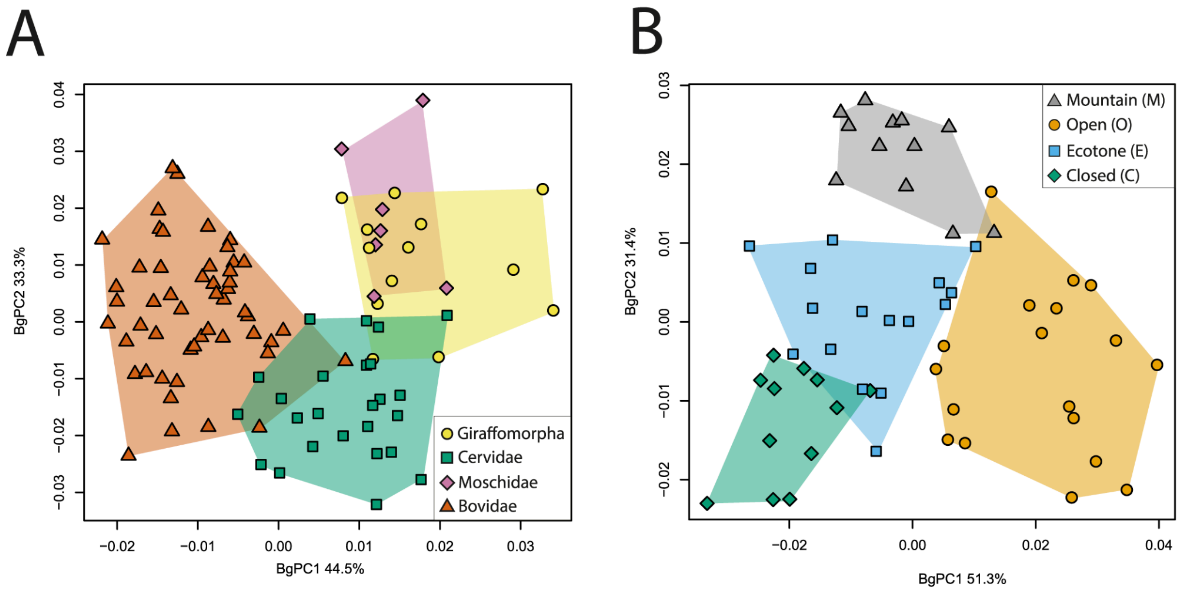

cross-validated classification results in frequencies

	Bovidae	Cervidae	Giraffomorpha	Moschidae
Bovidae	41	7	1	2
Cervidae	4	19	1	1
Giraffomorphi	0	2	9	2
Moschidae	0	2	1	4

cross-validated classification result in %

	Bovidae	Cervidae	Giraffomorpha	Moschidae
Bovidae	80.3922	13.7255	1.9608	3.9216
Cervidae	16	76	4	4
Giraffomorphi	0	15.3846	69.2308	15.3846
Moschidae	0	28.5714	14.2857	57.1429

overall classification accuracy: 76.04167 %

Kappa statistic: 0.62903

cross-validated classification results in frequencies

	E	F	M	O
E	9	2	4	1
F	6	6	0	0
M	2	0	8	2
O	3	1	1	13

cross-validated classification result in %

	E	F	M	O
E	56.25	12.5	25	6.25
F	50	50	0	0
M	16.6667	0	66.6667	16.6667
O	16.6667	5.5556	5.5556	72.2222

overall classification accuracy: 62.06897 %

Kappa statistic: 0.48796

Supplementary Figure 6. bgPCA performed in this study. (A) On extant and extinct Pecora. (B) On extant ruminants. Mean Procrustes coordinates per species were used.

References used for the phylogeny :

Aiglstorfer, M., Mayda, S. and Heizmann, E.P.J. First record of late middle Miocene Moschidae from Turkey: Micromeryx and Hispanomeryx from Catakbağyaka (Muğla, SW Turkey). *Comptes Rendus Palevol* 17 (3): 178–188 (2018).

Barry, J-C. Fossil tragulids of the Siwalik Formations of southern Asia - Zitteliana Serie B - 32: 53 - 61 (2014).

Bibi, F. A multi-calibrated mitochondrial phylogeny of extant Bovidae (Artiodactyla, Ruminantia) and the importance of the fossil record to systematics. *BMC Evolutionary Biology* 13 (1): 166 (2013).

Croitor, R. and Toderash, I.K. *Plio-Pleistocene deer of Western Palearctic: taxonomy, systematics, phylogeny*. Institute of Zoology of the Academy of Sciences of Moldova, Chișinău (2018).

Hassanin, A., Delsuc, F., Ropiquet, A., Hammer, C., Jansen van Vuuren, B., Matthee, C., Ruiz-Garcia, M., Catzeflis, F., Areskoug, V., Nguyen, T.T. and Couloux, A. Pattern and timing of diversification of Cetartiodactyla (Mammalia, Laurasiatheria), as revealed by a comprehensive analysis of mitochondrial genomes. *Comptes Rendus Biologies* 335 (1): 32–50 (2012).

Ghassemi-Khademi, T. and Madjdzadeh, S.M. Phylogenetic Relationships within the tribe Hippotragini (Antilopinae: Bovidae) Based on Mitochondrial Genome. 12 (3, 2019).

Mennecart, B., DeMiguel, D., Bibi, F., Rössner, G.E., Métais, G., Neenan, J.M., Wang, S., Schulz, G., Müller, B. and Costeur, L. Bony labyrinth morphology clarifies the origin and evolution of deer. Scientific Reports 7 (1): 13176 (2017).

Mennecart, B., Zoboli, D., Costeur, L. and Pillola, G.L. On the systematic position of the oldest insular ruminant *Sardomeryx oschiriensis* (Mammalia, Ruminantia) and the early evolution of the Giraffomorpha. *Journal of Systematic Palaeontology* 17 (8): 691–704 (2019).

Mennecart, B., Guignard, C., Dziomber, L., Schulz, G., Müller, B. and Costeur. Allometric and Phylogenetic Aspects of Stapes Morphology in Ruminantia (Mammalia, Artiodactyla). Frontiers in Earth Science 8: 176 (2020).

Mennecart, B., Dziomber, L., Aiglstorfer, M., Bibi, F., DeMiguel, D., Fujita, M., Kubo, M.O., Laurens, F., Meng, J., Métais, G., Müller, B., Ríos, M., Rössner, G.E., Sánchez, I.M., Schulz, G., Wang, S. and Costeur, L. Ruminant inner ear shape records 35 million years of neutral evolution (2022).

Ríos, M., Sánchez, I.M. and Morales, J. A new giraffid (Mammalia, Ruminantia, Pecora) from the late Miocene of Spain, and the evolution of the sivathere-samothere lineage. *PLOS ONE* 12 (11): e0185378 (2017).

Sánchez, I.M., Cantalapiedra, J.L., Ríos, M., Quiralte, V. and Morales, J. Systematics and Evolution of the Miocene Three-Horned Palaeomerycid Ruminants (Mammalia, Cetartiodactyla). PLOS ONE 10 (12): e0143034 (2015).
