# Supplementary material for: The differentiated impacts and constraints of allometry, phylogeny, and environment on the ruminants’ ankle bone
Source: Commun Biol. 2025 Mar 18;8:456. doi: 10.1038/s42003-025-07898-z (PMC11920208; doi:10.1038/s42003-025-07898-z)
Supplement: Supplementary file 3 — Description of Additional Supplementary Materials [file 42003_2025_7898_MOESM3_ESM.pdf]

Description of Additional Supplementary Files

File name: Supplementary Data

Description: Excel file including the detailed lists of individuals and the landmarking protocol used in this study
