# Supplementary material for: The differentiated impacts and constraints of allometry, phylogeny, and environment on the ruminants’ ankle bone
Source: Commun Biol. 2025 Mar 18;8:456. doi: 10.1038/s42003-025-07898-z (PMC11920208; doi:10.1038/s42003-025-07898-z)
Supplement: Supplementary file 4 — Reporting Summary [file 42003_2025_7898_MOESM4_ESM.pdf]

nature portfolio

Corresponding author(s): Pierre Orgebin

Last updated by author(s): Feb 10, 2025

Reporting Summary

Nature Portfolio wishes to improve the reproducibility of the work that we publish. This form provides structure for consistency and transparency in reporting. For further information on Nature Portfolio policies, see our Editorial Policies and the Editorial Policy Checklist.

Statistics

For all statistical analyses, confirm that the following items are present in the figure legend, table legend, main text, or Methods section.

n/a	Confirmed	
☐	☒	The exact sample size (n) for each experimental group/condition, given as a discrete number and unit of measurement
☒	☐	A statement on whether measurements were taken from distinct samples or whether the same sample was measured repeatedly
☐	☒	The statistical test(s) used AND whether they are one- or two-sided<br>*Only common tests should be described solely by name; describe more complex techniques in the Methods section.*
☐	☒	A description of all covariates tested
☐	☒	A description of any assumptions or corrections, such as tests of normality and adjustment for multiple comparisons
☐	☒	A full description of the statistical parameters including central tendency (e.g. means) or other basic estimates (e.g. regression coefficient) AND variation (e.g. standard deviation) or associated estimates of uncertainty (e.g. confidence intervals)
☐	☒	For null hypothesis testing, the test statistic (e.g. F, t, r) with confidence intervals, effect sizes, degrees of freedom and P value noted<br>*Give P values as exact values whenever suitable.*
☒	☐	For Bayesian analysis, information on the choice of priors and Markov chain Monte Carlo settings
☒	☐	For hierarchical and complex designs, identification of the appropriate level for tests and full reporting of outcomes
☒	☐	Estimates of effect sizes (e.g. Cohen's d, Pearson's r), indicating how they were calculated

Our web collection on statistics for biologists contains articles on many of the points above.

Software and code

Policy information about availability of computer code

Data collection	Artec Studio 17; Faro RevEng®2022.3; AVIZO 9.0; MeshLab® 1.3.3; Landmark Editor® 3.0.0.6
Data analysis	MorphoJ® 1.06b; R® 4.2.2; PAST® 2.17c

For manuscripts utilizing custom algorithms or software that are central to the research but not yet described in published literature, software must be made available to editors and reviewers. We strongly encourage code deposition in a community repository (e.g. GitHub). See the Nature Portfolio guidelines for submitting code & software for further information.

Data

Policy information about availability of data

All manuscripts must include a data availability statement. This statement should provide the following information, where applicable:

- Accession codes, unique identifiers, or web links for publicly available datasets
- A description of any restrictions on data availability
- For clinical datasets or third party data, please ensure that the statement adheres to our policy

Supplementary figures as well as the references used for the topology of the phylogeny and the results of statistical tests are available in the Supplementary Information pdf file.
The detailed lists of individuals and the landmarking protocol can be found in the Supplementary Data file.
All materials from which shape data are generated are housed in museum collections. 3D reconstructed models of astragali are either published and open access in

MorphoMuseuM (https://morphomuseum.com/) or will be.
The codes generated to conduct this study and files needed to run them, are available in the Zenodo repository https://doi.org/10.5281/zenodo.14844269

Research involving human participants, their data, or biological material

Policy information about studies with human participants or human data. See also policy information about sex, gender (identity/presentation), and sexual orientation and race, ethnicity and racism.

Reporting on sex and gender	Does not apply
Reporting on race, ethnicity, or other socially relevant groupings	Does not apply
Population characteristics	Does not apply
Recruitment	Does not apply
Ethics oversight	Does not apply

Note that full information on the approval of the study protocol must also be provided in the manuscript.

Field-specific reporting

Please select the one below that is the best fit for your research. If you are not sure, read the appropriate sections before making your selection.

☐ Life sciences ☐ Behavioural & social sciences ☒ Ecological, evolutionary & environmental sciences

For a reference copy of the document with all sections, see nature.com/documents/nr-reporting-summary-flat.pdf

Ecological, evolutionary & environmental sciences study design

All studies must disclose on these points even when the disclosure is negative.

Study description	Using 3D geometric morphometrics, we conducted allometric and size-adjusted clade and ecomorphological analyses to assess the contribution of each of these three parameters to the morphological variation of the astragalus in ruminant artiodactyls.
Research sample	The dataset comprises 205 astragali belonging to 58 extant species and 51 extinct species of ruminants.
Sampling strategy	We selected specimens with the aim of covering as much of the group's diversity as possible in terms of phylogeny, size and habitat.
Data collection	143 individuals were surface-scanned with an Artec Space Spider surface scanner and reconstructed on Artec Studio 17. Two specimens from MNHN were digitized with an Aicon Smart Scan at Plateau de Morphométrie du Muséum MNHN, CNRS UMS 2700 OMSI. Four specimens were surface-scanned using a FARO Laser ScanArm and digitized on Faro RevEng®2022.3 software. 33 individuals (ZFMK, SMNS) were scanned with x-ray computed tomography at the Staatliches Museum für Naturkunde Stuttgart using a Bruker Skyscan 1272 equipped with a 20-100kV / 10W x-ray source. 23 individuals were digitized using a nanoCT system nanotom (phoenix X-ray, GE Sensing and Inspection Technologies GmbH, Wunstorf, Germany) hosted at the Department of Biomedical Engineering, University of Basel. Additionally, four ct-scanned individuals from the Yale Peabody Museum were added to the sample. Details of the origin and digitization method of each individual can be found in the Supplementary Data file.
Timing and spatial scale	Data collected between 2017-2023
Data exclusions	Not all data was used in all analyses. Subsample were geenrated depending on the question. We used mean values per species. Mean values were computed with R.
Reproducibility	The analyses were repeated many times.
Randomization	Specimens were assigned to habitat categories based on literature.
Blinding	NA

Did the study involve field work? ☐ Yes ☒ No

Reporting for specific materials, systems and methods

We require information from authors about some types of materials, experimental systems and methods used in many studies. Here, indicate whether each material, system or method listed is relevant to your study. If you are not sure if a list item applies to your research, read the appropriate section before selecting a response.

Materials & experimental systems

n/a	Involved in the study
☒	☐ Antibodies
☒	☐ Eukaryotic cell lines
☐	☒ Palaeontology and archaeology
☒	☐ Animals and other organisms
☒	☐ Clinical data
☒	☐ Dual use research of concern
☒	☐ Plants

Methods

n/a	Involved in the study
☒	☐ ChIP-seq
☒	☐ Flow cytometry
☒	☐ MRI-based neuroimaging

Palaeontology and Archaeology

Specimen provenance	GSPY, Geological Survey of Pakistan and Yale Peabody Museum, Muséum National d'Histoire Naturelle, Paris, France; Natural History Museum of United Kingdom, London, United Kingdom; Museo Nacional de Ciencas Naturales, Madrid, Spain; Naturhistorisches Museum Bern, Bern, Switzerland; Naturhistorisches Museum Basel, Basel, Switzerland; Musée cantonal de géologie de Lausanne, Lausanne, Switzerland; Naturhistorisches Museum Mainz, Mainz, Germany; Staatliches Museum für Naturkunde Stuttgart, Stuttgart, Germany; Museum Koenig Bonn, Bonn, Germany.
Specimen deposition	All materials from which shape data are generated are housed in museum collections. 3D reconstructed models of the bony labyrinths are either published and open access in MorphoMuseuM (https://morphomuseum.com/) or will be.
Dating methods	No new dates are provided

☒ Tick this box to confirm that the raw and calibrated dates are available in the paper or in Supplementary Information.

Ethics oversight	GSPY, Geological Survey of Pakistan and Yale Peabody Museum, Muséum National d'Histoire Naturelle, Paris, France; Natural History Museum of United Kingdom, London, United Kingdom; Museo Nacional de Ciencas Naturales, Madrid, Spain; Naturhistorisches Museum Bern, Bern, Switzerland; Naturhistorisches Museum Basel, Basel, Switzerland; Musée cantonal de géologie de Lausanne, Lausanne, Switzerland; Naturhistorisches Museum Mainz, Mainz, Germany; Staatliches Museum für Naturkunde Stuttgart, Stuttgart, Germany; Museum Koenig Bonn, Bonn, Germany.

Note that full information on the approval of the study protocol must also be provided in the manuscript.

Plants

Seed stocks	Does not apply
Novel plant genotypes	Does not apply
Authentication	Does not apply
